# Genomic and epigenomic analysis of high-risk prostate cancer reveals changes in hydroxymethylation and TET1

**DOI:** 10.18632/oncotarget.8220

**Published:** 2016-03-21

**Authors:** Lien Spans, Thomas Van den Broeck, Elien Smeets, Stefan Prekovic, Bernard Thienpont, Diether Lambrechts, R. Jeffrey Karnes, Nicholas Erho, Mohammed Alshalalfa, Elai Davicioni, Christine Helsen, Thomas Gevaert, Lorenzo Tosco, Karin Haustermans, Evelyne Lerut, Steven Joniau, Frank Claessens

**Affiliations:** ^1^ Molecular Endocrinology Laboratory, Department of Cellular and Molecular Medicine, University of Leuven, Campus Gasthuisberg, Leuven, Belgium; ^2^ Current address: Laboratory for Genetics of Malignant Disorders, Department of Human Genetics, University of Leuven, Campus Gasthuisberg, Leuven, Belgium; ^3^ Department of Urology, University Hospitals Leuven, Campus Gasthuisberg, Leuven, Belgium; ^4^ Vesalius Research Center, VIB, Leuven, Belgium; ^5^ Laboratory of Translational Genetics, Department of Oncology, University of Leuven, Campus Gasthuisberg, Leuven, Belgium; ^6^ Department of Urology, Mayo Clinic, Rochester, MN, USA; ^7^ Research and Development, GenomeDx Biosciences, Inc., Vancouver, BC, Canada; ^8^ Organ Systems, Department of Development and Regeneration, University of Leuven, Campus Gasthuisberg, Leuven, Belgium; ^9^ Laboratory of Experimental Radiotherapy, Department of Oncology, University of Leuven, Campus Gasthuisberg, Leuven, Belgium; ^10^ Translational Cell & Tissue Research, Department of Imaging and Pathology, University Hospitals Leuven, Leuven, Belgium; ^11^ PEARL Consortium

**Keywords:** high-risk prostate cancer, genomics, TET1, epigenetics, DNA hydroxymethylation

## Abstract

The clinical heterogeneity of prostate cancer (PCa) makes it difficult to identify those patients that could benefit from more aggressive treatments. As a contribution to a better understanding of the genomic changes in the primary tumor that are associated with the development of high-risk disease, we performed exome sequencing and copy number determination of a clinically homogeneous cohort of 47 high-risk PCas. We confirmed recurrent mutations in *SPOP*, *PTEN* and *TP53* among the 850 point mutations we detected. In seven cases, we discovered genomic aberrations in the *TET1* (Ten-Eleven Translocation 1) gene which encodes a DNA hydroxymethylase than can modify methylated cytosines in genomic DNA and thus is linked with gene expression changes. TET1 protein levels were reduced in tumor versus non-tumor prostate tissue in 39 of 40 cases. The clinical relevance of changes in TET1 levels was demonstrated in an independent PCa cohort, in which low TET1 mRNA levels were significantly associated with worse metastases-free survival. We also demonstrate a strong reduction in hydroxymethylated DNA in tumor tissue in 27 of 41 cases. Furthermore, we report the first exploratory (h)MeDIP-Seq analyses of eight high-risk PCa samples. This reveals a large heterogeneity in hydroxymethylation changes in tumor versus non-tumor genomes which can be linked with cell polarity.

## INTRODUCTION

Prostate cancer (PCa) is the most frequently diagnosed cancer among European men [1]. One of the most important clinical challenges is to identify those patients that will develop lethal PCa. To tackle this, nomograms have been developed to identify patients with the highest risk of harboring lethal forms of PCa (according to the D’Amico criteria), but even within the high-risk subgroup there is a large heterogeneity in terms of outcome [2]. Hence, new markers are needed to help identify the lethal forms of PCa within the high-risk population. Exome sequencing of large PCa cohorts has already revealed multiple somatic base pair substitutions [3–5]. The challenge now is to define the consequences of these mutations and to identify those changes which can serve as markers to classify patients according to disease aggressiveness [3, 4].

It is well known that patterns of DNA methylation can be profoundly altered in cancer, including PCa [6–10]. A global genomic hypomethylation together with the hypermethylation of specific gene promoters and CpG islands results in cancer-related changes in gene expression. Although DNA methylation was discovered decades ago, the mechanisms controlling its dynamics are only starting to being unraveled [11]. In 2009, TET1 (Ten-Eleven Translocation 1) was identified as a dioxygenase that converts 5-methylcytosine (5mC) to 5-hydroxymethylcytosine (5hmC) [12]. While 5hmC is an intermediate to DNA demethylation, it could also serve in its own right as a new epigenetic marker. Hence, genomic hydroxymethylation as well as demethylation could lead to changes in gene expression [13, 14].

The TET1 enzyme has not been discussed in previous PCa sequencing papers, although it has been associated with PCa or suggested to act as a tumor suppressor. Since the *TET1* gene was recurrently affected in our cohort of high-risk PCa (HRPC), and because of its role in epigenetics, we specifically focused on changes in TET1 and DNA hydroxymethylation in this paper.

## RESULTS

### Detection of point mutations using whole exome sequencing

**Figure 1:**
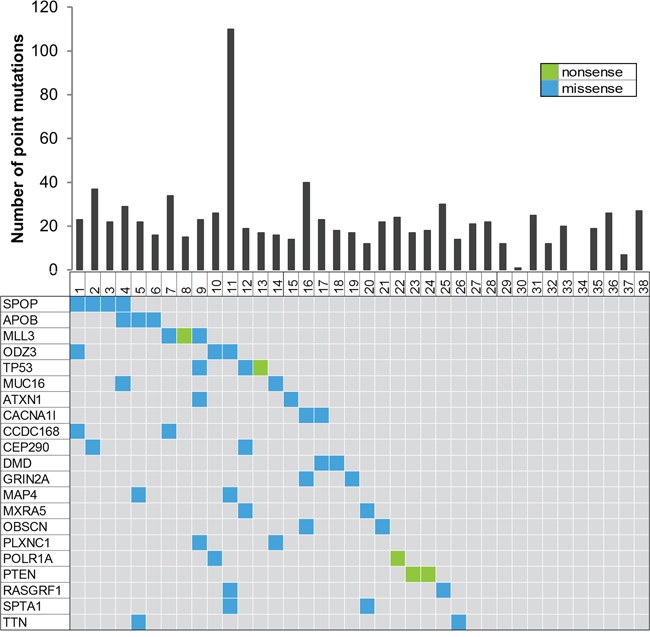
Summary of the whole exome sequencing The top of the figure shows a histogram representing the number of point mutations detected for each of the 38 prostate cancer samples. The bottom figure represents recurrently mutated genes, colored by the coding consequence of the mutation. Each column represents a tumor sample, and each row represents a gene.

Biopsies of 4 mm diameter were taken from 38 prostatectomy samples with HRPC (the clinical characteristics are shown in Supplementary Table 1 and summarized in Table 1). An overview of the sequencing alignment results is shown in Supplementary Table 2. Thirty-eight tumors harbored a median of 21 tumor-specific missense and nonsense single nucleotide variants (SNVs) (range 1 to 110 mutations). In total, we detected 850 SNVs in 736 different genes and 21 recurrently mutated genes (Figure 1, Supplementary Table 3). Of the 356 SNVs that were validated by Sanger sequencing or mass spectrometry genotyping, 77% were confirmed.

**Table 1:** Summary of clinical characteristics

Age at surgery (years)	
median (range)	64 (51-75)
Follow-up (months)	
median (range)	22 (14-45)
Pre-operative serum PSA (ng/ml)	
PSA < 20	33
PSA ≥ 20	5
Pathologic stage	
T2c	11
T3a	16
T3b	10
T4	1
Gleason score	
7	27
8	6
9	5
Surgical margin status	
Positive	11
Negative	27
Biochemical recurrence	
Positive	4
Negative	34

### Reduced 5hmC and TET1 is a common feature of high-risk PCa

**Figure 2:**
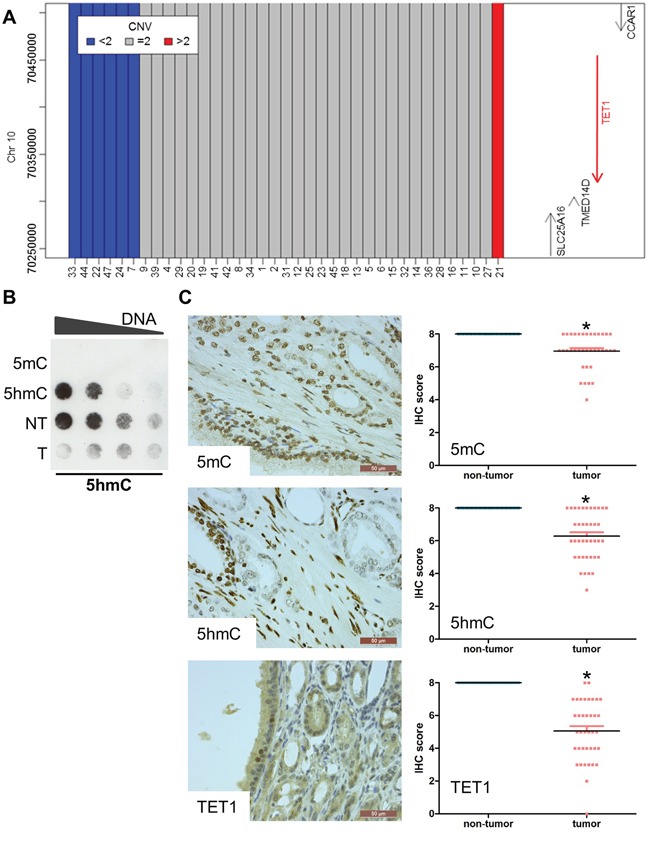
Reduced 5hmC and TET1 in prostate cancer **A.** SNP genotyping to detect copy number variants in the 10q23.1 region encompassing the *TET1* gene. Six samples lost one copy of the *TET1* gene. **B.** Dot blotting shows that the 5hmC level is decreased in tumor tissue. Genomic DNA isolated from tumor and non-tumor tissue of the patient with the *TET1* mutation (sample 25) was serially diluted. DNA containing only 5mC or 5hmC was used as negative and positive control respectively. **C.** Immunohistochemical stainings of 5mC, 5hmC and TET1. Magnification of the images is indicated by the scale bars. Stainings were performed on 41 HRPC samples. The scatter plots show the changes compared to non-tumor tissue. All scores were significantly different when comparing tumor with non-tumor tissue (p<0.05).

In one sample (sample 25), we detected an A1908S mutation in the catalytic domain of *TET1* as one of 30 mutated genes (Supplementary Figure 1). To further test for genomic aberrations, we determined whole genome copy number variations by SNP genotyping in 39 samples of HRPC. Much to our surprise, six samples lost one copy of the region encompassing the *TET1* locus, illustrating that this gene is recurrently affected in PCa (Figure 2A). Subsequently, we searched for changes in TET1 activity in PCa by analyzing the genomic 5hmC levels. A dot blot assay of the tumor DNA from the patient with the mutated *TET1* indeed contained less 5hmC than non-tumor tissue (Figure 2B).

Next, we used immunohistochemistry to analyze the 5mC, 5hmC and TET1 levels in a cohort of 40 HRPC samples largely overlapping with the cohort of samples for which we determined the exomes (Supplementary Table 6). Clearly, the signals of 5mC, 5hmC and TET1 in tumor tissue were lower than those in the adjacent non-tumor tissue of the same patient (Figure 2C). More specifically, 5mC was reduced in 25 of 40 samples, while 5hmC was reduced in 27 of 41 samples. These reductions were mainly due to a decreased intensity of the signals, since the number of cells that are positive for 5mC and 5hmC was similar in tumor and non-tumor tissue (Supplementary Figure 2). For TET1, a reduction in signal was seen in 39 of 40 samples. This reduction in global level was caused by a reduction in intensity as well as in the number of positive cells. For all of the seven samples with alterations in the *TET1* locus, a reduction in TET1 staining intensity was observed.

### Genome-wide mapping of 5mC and 5hmC

From our immunohistochemistry and dot blot data (Figure 2B), we know that overall nuclear 5hmC levels are lower in PCa versus non-tumor tissue, but nothing is known about the genomic distribution of 5hmC in PCa. We therefore determined the genome-wide 5mC and 5hmC distribution in seven PCa samples with wild type *TET1* genes. Immunoprecipitation of methylated versus hydroxymethylated DNA was followed by deep sequencing (MeDIP-Seq and hMeDIP-Seq). For each case, we compared DNA isolated from tumor and non-tumor prostate tissue.

**Figure 3:**
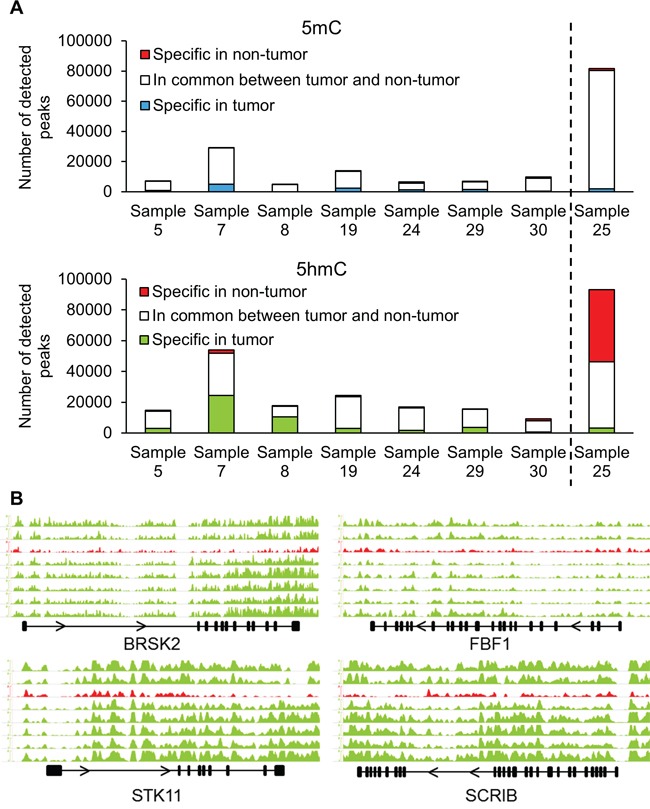
(h)MeDIP-Seq demonstrates changes in hydroxymethylation of cell polarity genes **A.** Immunoprecipitation of methylated and hydroxymethylated DNA followed by deep sequencing was performed on DNA isolated from the tumor and non-tumor tissues of eight patients. Sample 25 had the A1908S mutation in *TET1*. **B.** Distribution of 5hmC densities in the gene bodies of *BRSK2*, *STK11*, *FBF1* and *SCRIB* genes. The graph in red (third from top) represents the hydroxymethylation levels of sample 25 with the A1908S *TET1* mutation. Scale bars were equalized across all samples.

Figure 3A illustrates the divergence in numbers of methylation and hydroxymethylation peaks in tumor and non-tumor DNA across the different HRPC samples. It is important to note that similar read numbers of the DIP-seq were obtained (Supplementary Table 4). First of all, the well-known hypermethylation at the *GSTP1* locus, which serves as a validation of our assays, is also present in our dataset (Supplementary Figure 3). Overall, at the methylation level, there is a large inter-individual variability in numbers of detected methylation peaks (from 4655 to 28936). Despite these large inter-individual variations, there are substantial overlaps at the intra-individual level between tumor and non-tumor DNA (between 91 and 78%).

For the hydroxymethylated peaks, there is also a considerable overlap (> 75%) between tumor and adjacent tissue for five of the seven samples. Two samples had a more pronounced difference: sample 7 (50% overlap) and sample 8 (38% overlap) (Figure 3A). A more detailed localization of the peaks revealed that 5mC is mainly reduced in gene bodies and other intergenic regions of tumor tissue compared with non-tumor tissue (Supplementary Figure 4). Overall, however, while promoters in tumor tissue are hypermethylated, the reduction in 5hmC peaks is most apparent in promotor regions as well as gene bodies.

### (h)MeDIP-Seq of sample with mutated *TET1*

We subsequently performed a (h)MeDIP-Seq analysis of the DNA from the patient whose tumor carried the TET1 mutation, and compared it with that of the seven other patients with wild type *TET1* genes. First of all, there are more methylation peaks in the tumor as well as non-tumor sample of this patient than in the other samples (+/− 80 000). There are also more hydroxymethylation peaks in the non-tumor DNA of this sample (>89000) than in the seven other samples (ranging from 7189 to 29625). Finally, there are far less hydroxymethylation peaks in this tumor sample (sample 25 in Figure 3) than in the non-tumor counterpart. This contrasts with the seven other samples where the numbers of hydroxymethylation peaks in tumor DNA are equal or higher than in non-tumor DNA (Figure 3A). These data suggest that the mutation affects the function of TET1, and corroborates the notion that TET1 is important for DNA demethylation, also in PCa.

When comparing (h)MeDIP-Seq data of tumor DNA from seven samples with wild type *TET1* versus one sample with the A1908S mutation using diffReps, we detected 33 differentially methylated regions and 933 differentially hydroxymethylated regions (Supplementary Table 5). As expected, the majority of differentially methylated regions (32/33) showed increased methylation in the tumor with mutant *TET1*, while most of the differentially hydroxymethylated regions (618/933) were hypohydroxymethylated compared to tumor samples with wild type *TET1*. The annotation of hydroxymethylated regions revealed that genes involved in the establishment of cell polarity were affected: *BRSK2, STK11, FBF1* and *SCRIB* all displayed a marked reduction in 5hmC (Figure 3B). The methylation status of these genes is shown in Supplementary Figure 5. Whole-transcriptome data of an independent cohort of 326 samples (GSE46691) was used to study the correlation between the mRNA expression of TET1 and the four other cell polarity genes [15]. Indeed, the mRNA expression of each gene was strongly correlated with TET1 expression: BRSK2 p=3.7e-7; STK11 p=5.8e-11; FBF1 p=2e-9; SCRIB p=1.9e-11. In other words, low expression of TET1, resulting in decreased hydroxymethylation, strongly correlates with low expression of these four genes involved in cell polarity.

### Differentially expressed genes in PCa with high versus low TET1

**Figure 4:**
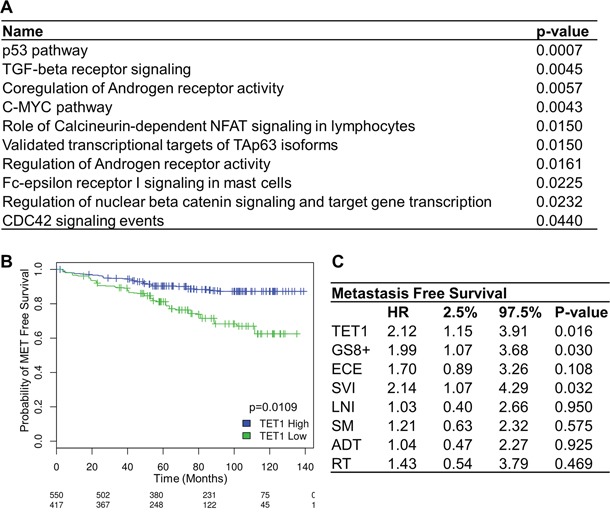
TET1 expression in prostate cancer **A.** EnrichR was used to obtain those pathways that contain downregulated genes in samples with a low TET1 mRNA expression level. **B.** Kaplan-Meier curve showing metastases-free survival in patients with high and low levels of TET1 expression. **C.** Multivariate metastases-free survival analysis using Cox's regression model. Abbreviations: GS8+, Gleason score 8 or above; ECE, extracapsular extension; SVI, seminal vesicle invasion; LNI, lymph node invasion; SM, surgical margins; ADT, adjuvant androgen-deprivation therapy; RT, adjuvant radiotherapy.

To compare the biological functions affected by TET1, we analyzed a larger set of retrospective samples using whole-transcriptome data, and compared samples with high TET1 expression versus samples with low TET1 expression levels. A training set of 1036 samples (GSE62667, GSE46691, GSE21032, and GSE41408) was used to select an optimal cutoff TET1 level to segregate patients with or without the development of metastases using logistic regression [15–19]. This cutoff of 0.14 was used to dichotomize patients of the testing set into groups with high and low TET1 mRNA expression. The testing set consisted of 235 patients from an independent cohort of the Mayo Clinic (GSE62116) [20]. In this cohort of PCa patients treated with radical prostatectomy, high-risk was defined as having a preoperative PSA > 20 ng/ml, Gleason score ≥ 8, pT3b or Mayo Clinic nomogram score ≥ 10. A list of genes of which the expression was differentially up- or downregulated in the group with low TET1 expression can be found in Supplementary Table 7. The expression of 449 genes was downregulated while 24 genes had higher expression levels. We used EnrichR to reveal more information about the pathways that are influenced by low levels of TET1 expression. Interestingly, the pathways regulating C-MYC and p53 were affected, as was the (co-)regulation of androgen receptor activity (Figure 4A).

### Expression of TET1 is associated with metastases-free survival

We next wondered whether the changes in 5hmC and TET1 may serve as a marker in the progression of PCa. Using the above stated testing set, Kaplan-Meier analysis showed that low mRNA expression levels of TET1 are significantly associated with worse metastases-free survival (p=0.0109) (Figure 4B). Multivariate regression analyses of the same cohort confirmed that TET1 mRNA expression is an independent predictor of metastases-free survival. With a hazard ratio of 2.12, patients with low TET1 mRNA expression levels have a doubled risk at developing metastases compared to patients with high TET1 levels (Figure 4C). Moreover, TET1 is as powerful as high Gleason score or seminal vesicle invasion in predicting bad prognosis.

## DISCUSSION

### Defining mutations in high-risk primary prostate cancer

Exome sequencing of 38 HRPC tumors revealed recurrent mutations in *SPOP*, *TP53*, *PTEN* and *MLL3* with four, three, three and two mutations respectively. This is in agreement with the reported frequencies of 13%, 6%, 4% and 8% in *SPOP, TP53, PTEN* and *MLL3* [3, 21]. In addition to these known recurrently mutated genes, we found 17 more genes that are mutated in at least two samples (Figure 1). Although some of these genes can easily be linked with cancer biology (e.g. *ATXN1* and *PLXNC1*), for others no such association has been reported (e.g. *APOB*). Clearly, the more samples sequenced, the more likely rare mutations will be picked up. The fact that this is also true in our HRPC cohort illustrates the genetic heterogeneity of this disease, and at the same time, presents opportunities for precision medicine as well. However, further investigations need to determine which of these mutations could have the potential to become drug targets [5, 22].

### Loss of TET1 in high-risk PCa

In this study we detected a mutation in TET1, the enzyme that converts methylcytosines into hydroxymethylcytosines as an intermediate in DNA demethylation (reviewed in Branco *et al.*) [23, 24]. *TET1* is affected in 7 out of the 47 HRPC cases we examined thus far: one case has a point mutation and six other cases lost one copy of the region containing the *TET1* gene. Because of the increasing importance of methylation, demethylation and hydroxymethylation in cancer development, we have focused on the 5mC and 5hmC levels and genome-wide changes in HRPC.

### Globally reduced hydroxymethylation in high-risk PCa

We first analyzed 5mC and 5hmC levels in 40 HRPC samples by immunohistochemistry. This demonstrated an important decrease in hydroxymethylation levels in tumor versus adjacent non-tumor tissue. This reduction was even more pronounced than the genome-wide reduction of methylation. Decreased DNA hydroxymethylation and downregulation of TET1 based on immunohistochemical stainings has been described before in small cohorts of primary PCa [6, 8, 25]. In our larger cohort of only HRPC, both the relative intensity as well as the number of TET1 positive cells was reduced.

The global reduction in 5hmC in PCa can have different causes. In our cohort, one sample has a mutation and six samples have a deletion in the 10q21.3 region containing the *TET1* gene. Alternatively, TET1 enzymatic activity can be inhibited by accumulated metabolites resulting from mutations in isocitrate dehydrogenase, fumarate hydratase or succinate dehydrogenase [26]. However, thus far no such mutations have been described in PCa. Moreover, changed activity of DNA methyltransferases, deaminases and base-excision repair enzymes will affect 5mC, thus affecting 5hmC levels [27]. The TET1 expression or activity can be influenced by many factors including vitamin C and O-linked β-N-Acetylglucosamine transferase (OGT) [28]. Importantly, Itkonen *et al.* recently reported a correlation between OGT levels and high Gleason score, pT/N status and biochemical recurrence [29]. It is therefore tempting to speculate that also in PCa, *TET1* links metabolism with epigenetic signaling.

### Reduced hydroxymethylation of genes in the cell polarity pathway

We report here the first determination of the specific genomic sites of 5mC as well as 5hmC in HRPC, by deep sequencing eight tumor and non-tumor genomes of our cohort. The selection of these samples was independent of tumor size or clinical characteristics but merely defined by the presence of sufficient DNA for the (h)MeDIP analyses. The well-known cancer-specific hypermethylation of *GSTP1* [30] was also present (Supplementary Figure 3), validating our analyses. Similar to what has been described for methylation, 5hmC is reduced genome-wide while site-specific levels are increased. In our samples, we detected a reduced hydroxymethylation in the STK11, SCRIB, FBF1 and BRSK2 genes. These four genes are required for the establishment of epithelial cell polarity. The expression of SCRIB is frequently lost in more advanced tumors and this downregulation results in disrupted cell polarity, amongst others in breast and colorectal tumors [31, 32]. STK11 is a known tumor suppressor that regulates cell polarity by remodeling the actin cytoskeleton. Loss of STK11 expression increases migration and invasion in breast cancer, while a STK11 knock-out predisposed mice to prostatic intraepithelial neoplasia [33, 34]. The importance of FBF1 and BRSK2 in tumor initiation or progression has yet to be shown, but it is known that these genes are required for the establishment of epithelial cell polarity [35].

### Site-specific hydroxymethylation changes

The detection of site-specific changes in hydroxymethylation evokes the important questions on how TET1 activity can be directed and whether 5hmC can act as a specific epigenetic mark involved in chromatin organization and transcriptional regulation. Takai *et al.* recently showed that 5hmC is an epigenetic mark that activates gene expression in glioblastoma [36]. Furthermore, it has been shown that the TET1 mediated DNA hydroxymethylation affects the expression of tissue inhibitors of metalloproteinases (TIMPs) in breast cancer, and the WNT pathway in colon cancer [7, 37]. In PCa, we could not detect any changes in TIMPs (data not shown). This implies that TET1 can have tissue-specific activities.

### Pathway changes in samples with low TET1 expression

Pathway annotation was performed on genes that were up- or downregulated in samples with low TET1 mRNA expression. An example of a gene that is upregulated is the long non-coding RNA MALAT1. Higher MALAT1 expression is correlated with higher Gleason score, higher tumor stage and castration-resistant PCa [38]. Moreover, MALAT1 is a crucial RNA cofactor of EZH2 as it is involved in EZH2-enhanced migration and invasion in castration-resistant PCa [39]. On the other hand, the expression of GSK3beta is downregulated in samples with low TET1 RNA expression. GSK3beta has potent tumor suppressor qualities and decreased function may elicit increased activity of androgen receptor signaling [40]. The link between androgen stimulation and the recruitment of the androgen receptor and TET1 has been recently shown by Dhiman and colleagues [41].

Clearly, these first characterizations of 5hmC in HRPC and the pathways they affect are exploratory. However, they reveal a large heterogeneity, indicating that much is yet to be learned about the role of TET1 in PCa. Correlations with changes in the transcriptomes and chromatin marks will more clearly identify which pathways can be affected and these in turn might become prognostic factors or therapeutic targets in the long run.

### Clinical relevance of 5hmC in high-risk PCa

Because of the crucial role of TET1 in the regulation of the (hydroxy-)methylation and the low levels of 5hmC in most of our HRPC samples, the clinical relevance of TET1 expression was further investigated. In a retrospective analysis of an independent PCa cohort, the level of TET1 expression was associated with metastases-free survival as illustrated in Figure 4C. From our data, we conclude that TET1 could be a prognostic marker in PCa which might help to select patients for more aggressive treatment modalities. Whether this is also true for the global changes in 5hmC which would be easier to asses by immunohistochemistry awaits further investigations.

### General conclusion

Whole exome sequencing was performed on 38 samples of a clinically homogeneous group of HRPC samples. We confirmed recurrent mutations in PCa-specific genes, but also identified many new genes not known to be mutated, like *TET1*. We discovered a strong decrease in DNA hydroxymethylation in HRPC compared to surrounding non-tumor tissue. Finally, the identification of TET1 mRNA expression levels as an independent predictor of metastasis-free survival indicates an important role for TET1 as well as for hydroxymethylation in PCa.

## MATERIALS AND METHODS

### Sample acquisition and analyses

Primary tumors were obtained from patients with clinical HRPC undergoing radical prostatectomy between 2011 and 2014. High-risk definition was based on preoperative serum PSA levels > 20 ng/mL or Gleason score ≥ 8 or a clinical stage of T2c or higher [2]. This study was conducted in accordance with the Declaration of Helsinki and the study protocol was approved by the UZ Leuven Ethical Committee. Matching germline DNA was derived from peripheral blood cells. Specimens were collected at the University Hospitals of Leuven within the PEARL consortium (ProstatE cAncer Research team Leuven). The clinical characteristics of the patients are described in Table 1 and Supplementary Table 1, an overview of the samples used for different experiments in Supplementary Table 6.

Fresh-frozen biopsies containing > 75% tumor content were used for whole exome sequencing. Tumor content was estimated by a pathologist specialized in uro-oncological diseases (E. Lerut). Exome capture was performed using the SeqCap EZ Exome version 3 kit (Roche), after which 100 bp paired-end sequences were generated with a HiSeq instrument. Sequencing data were aligned to hg19 with BWA and processed by Picard (http://picard.sourceforge.net) [42]. Aligned files were processed with GATK and included duplicate removal, local realignment around known indels and base quality recalibration [43]. On average, 123 million reads were sequenced per sample, with 87% of target bases covered at a depth of ≥ 20x. Somatic SNVs were detected by comparing tumor and paired normal exome sequences with MuTect and SomaticSniper [44, 45]. SNVs were annotated with SeattleSeq (http://snp.gs.washington.edu/SeattleSeqAnnotation137/index.jsp). We focused on missense and nonsense SNVs absent in dbSNP132 and only retained those SNVs present in > 10% of tumor reads and < 2% of non-tumor reads. Whole exome data has been deposited at the European Genome-phenome Archive (EGA, http://www.ebi.ac.uk/ega/) which is hosted at the EBI, under accession number EGAS00001001015.

Sequenom MassARRAY validation was performed according to the manufacturer's conditions and automated genotyping calls were generated using the MassARRAY RTTM software. A subset of SNVs was validated using PCR followed by Sanger sequencing.

### Copy number determination

Genome-wide SNP genotyping was performed using Illumina CytoSNP arrays on an iSCAN. Processing of DNA samples, hybridization, staining, scanning of the BeadChips, and primary data extraction were all performed according to the Illumina Infinium protocol at the Vesalius Research Center (Leuven, Belgium). GenomeStudio software was used for primary assessment of data and quality control assessment. ASCAT (Allele-Specific Copy number Analysis of Tumors) (version 2.1) was used to determine copy number alterations in solid tumors, while estimating and correcting for both tumor aneuploidy and infiltration of non-aberrant cells [46]. To identify significantly amplified or deleted regions, GISTIC (Genomic Identification of Significant Targets in Cancer) (version 2.0.1) was used [47].

### (h)MeDIP-Seq

Three microgram genomic DNA was used to enrich DNA containing 5mC and 5hmC with specific antibodies (Eurogentec and Active Motif). The DNA was fragmented to 100-500 bp using the Diagenode bioruptor, and transformed into libraries using the NEBNext kit (New England Biolabs). Following denaturation, DNA was incubated overnight at 4°C with 5 μl of anti-5mC antibody or 3 μl of anti-5hmC antibody in IP buffer (1x PBS, 0.5% BSA, 1 mM EDTA, 0.05% Triton X-100). The following day, 10 μl of protein A/G magnetic beads (Pierce) was added, and after two hours beads were washed five times using IP buffer. DNA was eluted in 30 μl by heating the beads for 10 minutes at 99°C. The eluted DNA was amplified by 14 cycles of PCR using barcoded Illumina primers (New England Biolabs) and purified. A single 50 bp sequence was determined using HiSeq2000. Uniquely mapping sequences were aligned to hg19 using Bowtie with --strata --best as parameters [48]. MACS2 was used as the peak-finding algorithm after *in silico* extension of the reads by 100 bp [49]. Differentially (hydroxy-) methylated regions were called using diffReps and annotated using GREAT [50, 51]. For the log2 fold change, a cutoff of 2.5 was taken. Integrative Genomics Viewer was used to visualize sequence reads [52, 53]. (h)MeDIP-Seq data has been deposited at the European Genome-phenome Archive (EGA, http://www.ebi.ac.uk/ega/) which is hosted at the EBI, under accession number EGAS00001001019.

### Detection of hydroxymethylated DNA

Genomic DNA was isolated using the GenElute Mammalian Genomic DNA Miniprep kit (Sigma-Aldrich). DNA containing 5mC and 5hmC (Active Motif) was used as control. Dot blot experiments were performed as described except that an equal volume of 2 M ammonium acetate was added to neutralize samples after denaturation [54]. An anti-5hmC antibody was used (Active Motif), 1:1000.

### Immunohistochemistry

Antigen recovery on paraffin-embedded sections was performed in citrate buffer pH 6 for 20 minutes, followed by staining on the Bond Max Autostainer (Leica). The antibodies used are 5mC (1:1000, Eurogentec), 5hmC (1:2000, Active Motif) and TET1 (1:200, Sigma). Scoring was performed as described before [55]. Briefly, the semi-quantitative system takes into account the proportion of positive cells (range 0-5) and the staining intensity (range 0-3). Both scores were summed, resulting in a score between zero and eight.

### TET1 mRNA expression in high-risk prostate cancer

Whole-transcriptome data from 1271 PCa patients (GSE62667 [16]), GSE46691 [15], GSE21032 [17], GSE41408 [18, 19], GSE62116 [20]) was normalized and summarized to the Affymetrix core transcript cluster level using SCAN [56].

The correlations between TET1 mRNA expression and the expression of BRSK2, STK11, FBF1 and SCRIB were calculated using the GSE46691 cohort and using a generalized linear model to obtain p-values. Differentially expressed genes in this cohort were annotated using the NCI-Nature curated set in Enrichr [57].

To obtain the Kaplan-Meier (KM) curve, the samples were grouped into a training set and testing set, where [15–19] made up the training set (n = 1036) and [20] was used as the validation set of this case-cohort study design (n = 235). An optimal cutoff was selected by maximizing sensitivity and specificity (optimal.cutpoint 1.1-3 package). KM and multivariable survival analysis using Cox's regression model (survival 2.37-7 package) was performed on the validation dataset using groups defined by high and low TET1 mRNA expression. Significance between the two groups was assessed with the log-ranked test. All statistical analyses were performed using R 3.02.

### Statistical analysis

Analyses with student's T-test were done by Graphpad Prism. *P* < 0.05 was regarded as threshold value for statistical significance.

## SUPPLEMENTARY FIGURES AND TABLES












