# Supplementary material for: Genomic and epigenomic analysis of high-risk prostate cancer reveals changes in hydroxymethylation and TET1
Source: Oncotarget. 2016 Mar 21;7(17):24326–38. doi: 10.18632/oncotarget.8220 (PMC5029704; doi:10.18632/oncotarget.8220)
Supplement: Supplementary file 2 [file oncotarget-07-24326-s002.pdf]

SUPPLEMENTARY FIGURES AND TABLES

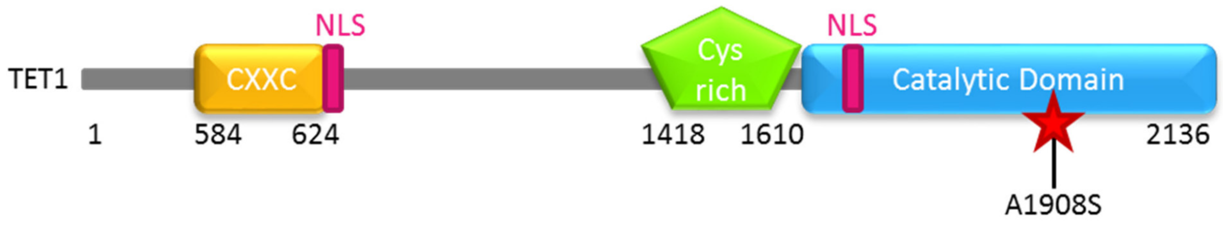

Supplementary Figure S1: Schematic diagram of full length TET1. NLS: nuclear localization signal; Cys-rich: cysteine-rich.

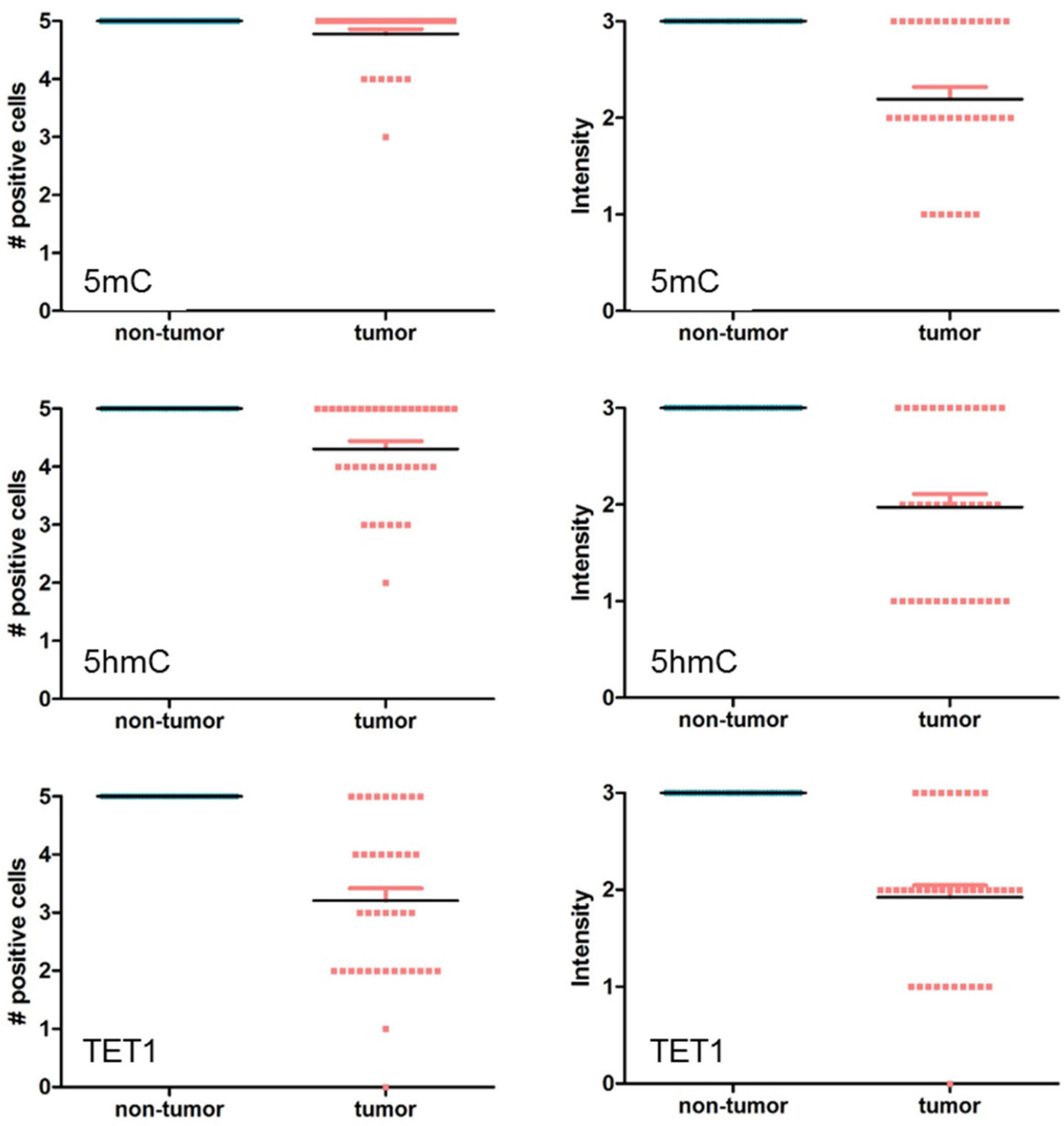

Supplementary Figure S2: Scoring of the 5mC, 5hmC and TET1 immunohistochemical stainings for the proportion of positive cells and the intensity of the staining. The maximum score for proportion of positive cells is 5, for intensity 3.

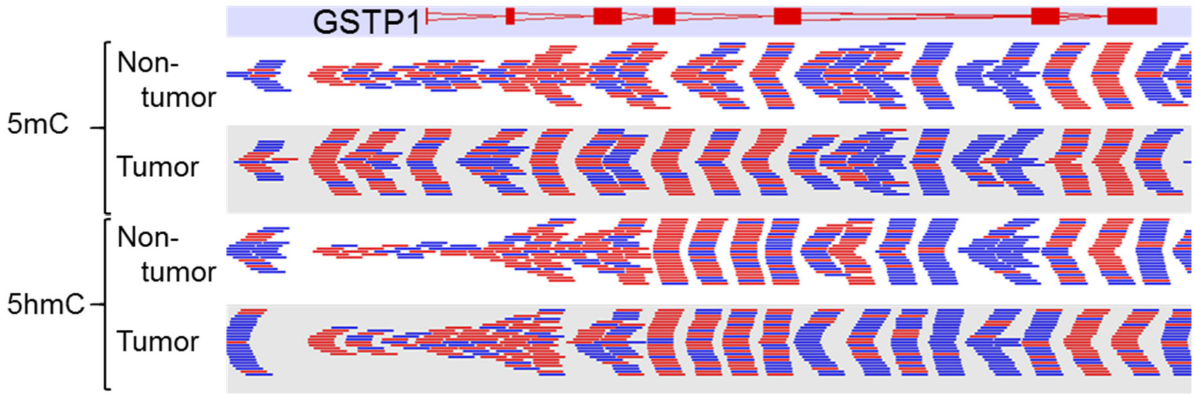

Supplementary Figure S3: Summary of (h)MeDIP-Seq reads around the GSTP1 gene. Reads of all samples were merged into one figure. Cancer-specific hypermethylation of the GSTP1 promoter is shown.

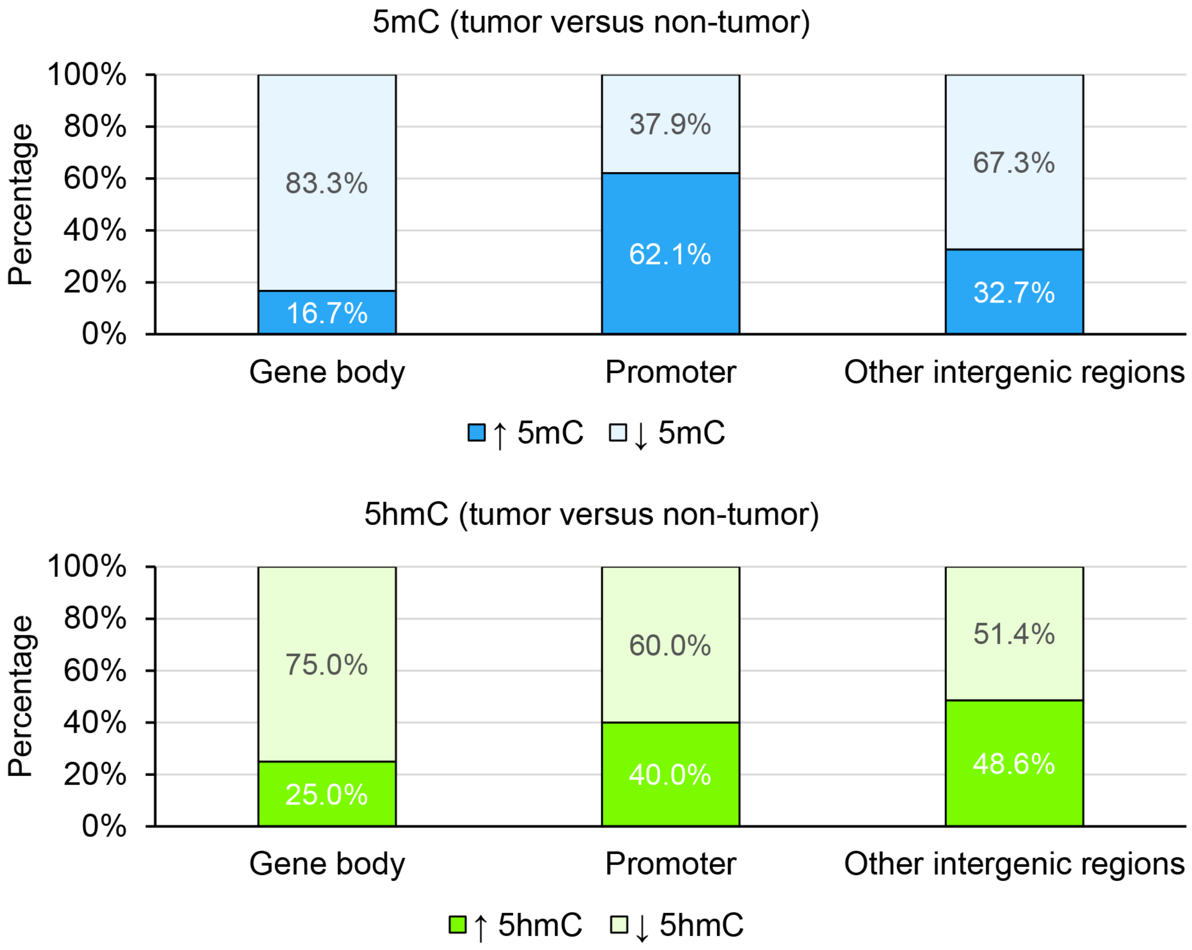

Supplementary Figure S4: Location of differential peaks called with diffReps. Graphs show whether peaks that are different in tumor compared to non-tumor are located in the gene body, promoter or other intergenic regions for 5mC (top) and 5hmC (bottom).

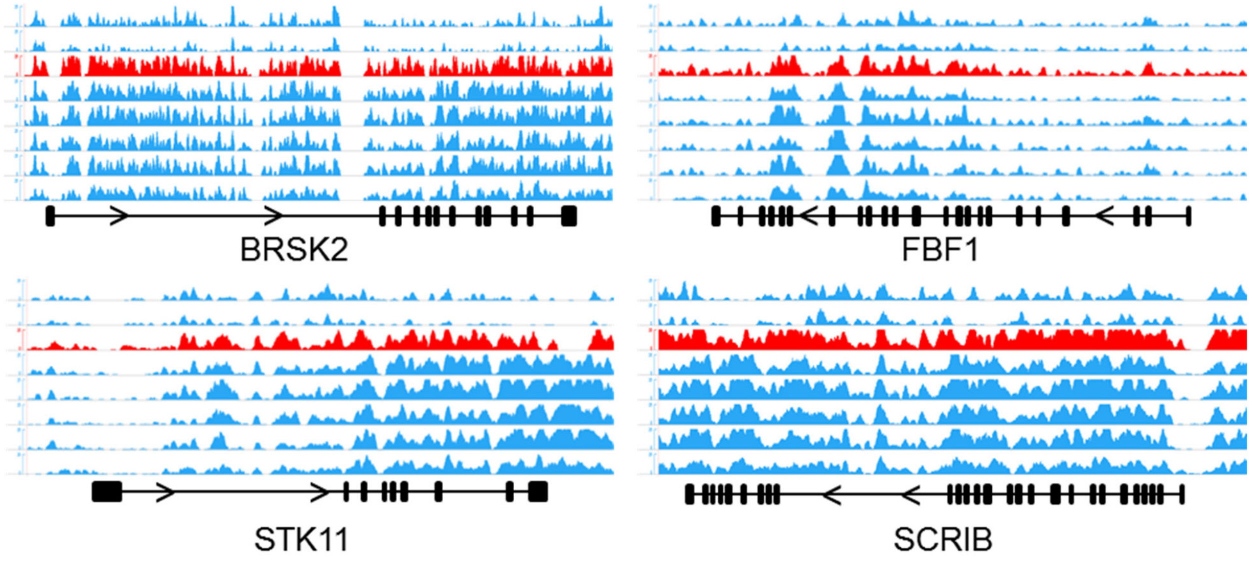

Supplementary Figure S5: Distribution of 5mC densities in the gene body of *BRSK2*, *STK11*, *FBF1* and *SCRIB* genes. The graph in red (third from top) represents the methylation levels of sample 25 with the A1908S TET1 mutation.

Supplementary Table S1: Clinical characteristics

See Supplementary File 1

Supplementary Table S2: Summary of alignment results over 78 exome samples

	Average	St. Dev.	Minimum	Maximum
Total number of reads (Mbp)	123.78	45.86	49.25	266.24
Number of high quality aligned bases (Gbp)	10.46	3.49	4.30	18.95
Mean read length	101	0	101	101
Percentage targets with zero coverage	2.33	1.25	1.67	10.00
Percentage of target bases covered at least 2x	96.25	1.71	86.15	97.60
Percentage of target bases covered at least 10x	92.93	2.36	80.23	95.33
Percentage of target bases covered at least 20x	87.00	4.80	73.34	93.37
Percentage of target bases covered at least 30x	77.84	9.38	49.18	91.31

Supplementary Table S3: Somatic mutations in 38 high-risk prostate tumor-normal pairs

See Supplementary File 2

Supplementary Table S4: Summary of alignment results over 32 DIP-Seq samples

	Average	St. Dev.	Minimum	Maximum
Total number of reads (Mbp)	25.19	10.40	14.11	51.25
Number of high quality aligned bases (Gbp)	1.05	0.29	0.70	1.80
Mean read length	50	0	50	50

Supplementary Table S5: List of differentially methylated and hydroxymethylated regions when comparing one sample with mutated TET1 to seven samples with wild type TET1

See Supplementary File 3

Supplementary Table S6: Overview of samples used for different experiments

See Supplementary File 4

Supplementary Table S7: List of genes that are differentially expressed in samples with low TET1 mRNA expression compared to samples with high TET1 mRNA expression

See Supplementary File 5
