# Supplementary material for: Genomic and epigenomic analysis of high-risk prostate cancer reveals changes in hydroxymethylation and TET1
Source: Oncotarget. 2016 Mar 21;7(17):24326–38. doi: 10.18632/oncotarget.8220 (PMC5029704; doi:10.18632/oncotarget.8220)
Supplement: Supplementary file 5 [file oncotarget-07-24326-s005.doc]

**Supplementary table 6.** Overview of samples used for different experiments.
